# Supplementary material for: Negative Regulatory Loop between Microphthalmia-Associated Transcription Factor (MITF) and Notch Signaling
Source: Int J Mol Sci. 2019 Jan 29;20(3):576. doi: 10.3390/ijms20030576 (PMC6387231; doi:10.3390/ijms20030576)
Supplement: Supplementary file 1 [file ijms-20-00576-s001.pdf]

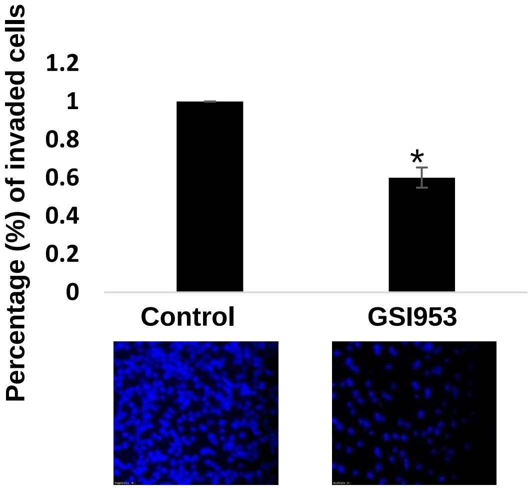

Supplementary figure S1. Notch signaling promotes melanoma invasion. WM3314 cells were treated with 20μM PF3084014 and an invasion assay was conducted. Graph demonstrates proportion (%) of invading cells from the total seeded cells, and a representative image of invading cells is shown for each treatment in the lower panel; blue is DAPI nuclear staining. Error bars represent ± SEM, ∗ indicates $p < 0.05$ ($n = 4$).
